# Substance use, mental health and weight‐related behaviours during the COVID‐19 pandemic in people with obesity

**DOI:** 10.1111/cob.12440

**Published:** 2021-02-04

**Authors:** Jaime P. Almandoz, Luyu Xie, Jeffrey N. Schellinger, M. Sunil Mathew, Nora Bismar, Ashley Ofori, Sachin Kukreja, Benjamin Schneider, Denise Vidot, Sarah E. Messiah

**Affiliations:** ^1^ Department of Internal Medicine, Division of Endocrinology University of Texas Southwestern Medical Center Dallas Texas USA; ^2^ Department of Epidemiology, Human Genetics and Environmental Sciences University of Texas Health Science Center, School of Public Health Dallas Texas USA; ^3^ Center for Pediatric Population Health Children's Health System of Texas and UT Health School of Public Health Dallas Texas USA; ^4^ Minimally Invasive Surgical Associates Dallas Texas USA; ^5^ Department of Surgery University of Texas Southwestern Medical Center Dallas Texas USA; ^6^ School of Nursing University of Miami Miami Florida USA; ^7^ Present address: Paul M. Bass Administrative and Clinical Center Dallas Texas USA

**Keywords:** COVID‐19, ethnicity, mental health, obesity, substance use

## Abstract

Studies have shown the negative impact of COVID‐19 lockdown orders on mental health and substance use in the general population. The aim of this study was to examine the impact of the COVID‐19 pandemic onsubstance use, mental health and weight‐related behaviors in a sample of adults with obesity after lockdown orders were lifted (June‐September 2020). A retrospective medical chart review identified patients with obesity from one university‐based obesity medicine clinic, and two metabolic and bariatric surgery (MBS) practices. Patients who completed an online survey from June 1, 2020 to September 30, 2020 were included. The primary outcome measure was substance use (various drugs, alcohol, tobacco). Substance use and mental health survey questions were based on standardized, validated instruments. A total of 589 patients (83.3% female, mean age 53.6 years [SD 12.8], mean BMI 35.4 [SD 9.1], 54.5% Non‐Hispanic white, 22.3% post‐MBS) were included. Seventeen patients (2.9%) tested positive for SARS‐CoV‐2 and 13.5% reported symptoms. Nearly half (48.4%) of the sample reported recreational substance use and 9.8% reported increased use since the start of the pandemic. There was substantial drug use reported (24.3% opioids, 9.5% sedative/tranquilizers, 3.6% marijuana, and 1% stimulants). Patients who reported stockpiling food more (adjusted Odds Ratio [aOR] 1.50, 95% CI 1.03‐2.18), healthy eating more challenging (aOR 1.47, 95% CI 1.01‐2.16), difficulty falling asleep (aOR 1.64, 95% CI 1.14‐2.34), and anxiety (aOR 1.47, 95% CI 1.01‐2.14) were more likely to report substance use versus non‐users. Results here show that the COVID‐19 pandemic is having a deleterious impact on substance use, mental health and weight‐related health behaviors in people with obesity regardless of infection status.

## 1 INTRODUCTION

The coronavirus disease 2019 (COVID‐19) pandemic has been associated with mental health challenges related to public health recommendations, including social distancing and stay‐at‐home orders, as well as morbidity and mortality.^1^ Population surveys have shown a substantial increase in self‐reported anxiety and depression in the United States^2^, ^3^ and other countries^4^, ^5^, ^6^ between April and June 2020 compared with the same period in 2019. Surveys show that over 40% of respondents reported at least one mental health condition, including anxiety or depression (30.9%), symptoms of a trauma‐ or stressor‐related disorder (26.3%), and having started or increased substance use (13.3%) to cope with the COVID‐19 pandemic.^1^

Previous research suggests there is a relationship between food and beverage over‐consumption and substance use disorders^7^ as palatable foods containing high concentrations of sugar and fat activate the same dopamine pathways in the brain's reward system as addictive drugs.^8^ Some studies suggest that people with obesity have a higher risk of substance use disorders following metabolic and bariatric surgery (MBS) due to the “addiction transfer” phenomenon.^9^ There are limited studies on the relationship between substance use disorder and obesity. Some data show no relationship between illicit drug use disorders and BMI^10^ and that people with obesity have a lower lifetime risk of substance use disorder.^11^

There is evidence that stress increases the risk of developing substance use disorders and that there is a bidirectional relationship between stress and obesity, through cognitive and physiological mechanisms.^12^, ^13^ During the COVID‐19 pandemic, our anecdotal clinical experiences with our patients suggested that substance misuse may be occurring more frequently in this group than was previously thought. Therefore, the goal of this study was to evaluate the relationship between the pandemic and the misuse of substances, including alcohol, recreational drugs and prescription medications among people with obesity. Based on our clinical experiences, we hypothesized that patients with obesity would report increased substance use in response to the stress and disruption of the COVID‐19 pandemic.

## 2 METHODS

### 2.1 Design

A retrospective chart review identified patients with obesity from an academic healthcare system's obesity medicine clinic, MBS clinic, and a community‐based MBS practice.

### 2.2 Procedure

An online non‐anonymous survey was implemented from 1 June 2020 to obtain information about the COVID‐19 pandemic's impact on patients with obesity. This was approximately 2 months after the Governor of Texas mandated stay‐at‐home orders (31 March 2020). The University of Texas Health System Institutional Review Board approved the study. Patients were asked to respond to a 15 minutes survey about the COVID‐19 pandemic's effect on their health and lifestyle behaviours. Participants signed an online consent and authorized contact for follow up information. Study data were collected and managed using Research Electronic Data Capture (REDCap)^14^, ^15^ electronic data capture tools hosted at the UT Southwestern Medical Center.

### 2.3 Measures

Participants were queried on a variety of areas including demographics and the impact of the COVID‐19 pandemic on their lifestyle behaviours, substance use, and physical and mental health. The primary dependent variable of interest was substance use (Y/N), and primary independent variables were weight‐related behaviours and psychological factors, such as anxiety and depression. Covariates include age, gender, education, BMI and COVID‐19 infection.

### 2.4 Demographics

Demographic questions were based on the validated instrument, Behavioural Risk Factor Surveillance System (BRFSS).^16^ Respondents were asked their gender, race/ethnicity, age, marital status, education level, basic anthropometrics (height/weight), household income and occupant information. ZIP code and county were gathered to cross reference with local COVID‐19 infection rates.

### 2.5 Employment

The COVID‐19 pandemic has had a significant impact on the US economy and employment rates.^1^ The survey assessed changes in employment, including job loss (Have you lost your job because of COVID‐19?) and decrease in working hours (Have you had a reduction in job hours because of COVID‐19?). Essential worker designation was also determined (Are you considered an essential employee and required to physically go to work? You are NOT allowed to work remotely from home) and if they were working remotely (Are you CURRENTLY working remotely from home?).

### 2.6 COVID‐19

COVID‐19 related questions focused on infection of the subject or family members with the virus (Have any of your family members tested positive for COVID‐19?), history of testing (Have you been tested for COVID‐19?), difficulty getting tested (Have you wanted to get tested for COVID‐19 but found it difficult to do so?), or COVID‐19 symptoms (Have you had any symptoms associated with COVID‐19?). All participants were asked about existing chronic medical conditions, which would put them at greater risk for more severe COVID‐19 complications.

### 2.7 Lifestyle behaviours

The COVID‐19 pandemic has resulted in considerable changes to everyday life. Survey respondents were asked about the frequency that they were leaving the house and social activities, which ranged from staying completely isolated at home to attending large social gatherings. A variety of Likert scale questions required respondents to compare their lifestyle behaviours before and since the COVID‐19 pandemic (How often do you go out to the grocery store to shop for food?) (Do you stockpile food due to COVID‐19?). Questions related to their health included changes to exercise, healthy habits, and the quality and quantity of food consumption (Has it been more challenging to stick with your healthy eating pattern due to the available food options in your local grocery store?) (Since COVID‐19, has your consumption of the following foods [fast foods, fresh fruits, pizza, processed meats, red meat, restaurant foods, snacks/potato chips, soda/sugar sweetened beverages, vegetables] changed?) (Do you COOK more or less due to COVID‐19?) (Do you BAKE more or less due to COVID‐19?) (Have you increased ordering restaurant to‐go/delivery foods to avoid going to the grocery store?). Additionally, food security was evaluated using the validated 6‐item U.S. Adult Food Security Survey Module.^17^

### 2.8 Depression

Depressive symptoms were assessed in survey respondents with the validated 16‐item Quick Inventory of Depressive Symptomatology (QIDS‐SR_16_).^18^ They were queried on variety of elements, such as sleep, appetite, anxiety, and so forth, and how these had changed over the prior 7 days.

### 2.9 Substance use

Substance use behaviours was assessed by National Epidemiologic Survey on Alcohol and Related Conditions (NESARC),^19^ which is the primary dependent variable of interest of this study. Participants reporting use of any illicit drugs, having more than four alcoholic beverages during the past 30 days, or current cigarette smoking were categorized as substance users and composed of 48.4% (N = 285) of the sample. Others were categorized as non‐users. To assess the change of these behaviours due to the pandemic, we asked the participants to report if they increased the use of any recreational substances, including alcohol and tobacco, since the COVID‐19 stay‐at‐home orders were issued.

## 3 STATISTICAL ANALYSIS

Descriptive analysis was performed for baseline characteristics including age, sex, race/ethnicities (non‐Hispanic white [NHW], non‐Hispanic black [NHB], Hispanic, and other) education, annual household income, BMI, and chronic medical conditions. COVID‐19 related information including test results (positive or negative), symptom (yes or no) and level of quarantine (from not going outside at all to going out as normal). Detailed information on substance use, including drug, alcohol, and tobacco use, was also reported. Pearson chi‐square tests were utilized to compare of the change of weight related behaviours and psychological factors, such as food stockpiling, stress eat, difficulty falling asleep, anxiety and depression, after COVID‐19 stay‐at‐home orders being lifted by substance use groups. Crude odds ratios and adjusted odds ratios were calculated for substance use (Y/N) and increased substance use since COVID‐19 (Y/N), respectively, using weight‐related behaviours and psychological factors of interest as independent variables. Adjusted logistic regression models also controlled for age, gender, race/ethnicity, education, BMI, prior MBS (Y/N) and COVID‐19 infection. We also compared substance used behaviours by ethnic groups via Person chi‐square tests. All statistical analyses were performed using SAS v9.4 (SAS Institute, Cary, North Carolina). 2‐sided *P* value <.05 is considered significant.

## 4 RESULTS

From 1 June 2020 to 3 November 2020, a total of 783 patients have enrolled in the COVID‐19 and obesity phase II study. We excluded those who did not complete the patient consent form (n = 7) or did not answer questions regarding alcohol use (n = 187). Thus, the final analytical sample included 589 patients (83.3% female, mean age 53.6 years, SD 12.8). More than a half (54.5%) were NHW, 21.5% were NHB, 19.8% were Hispanic and 4.2% identified as “other” (multiracial, Asian, etc.). The majority (57.8%) were college graduates, and more than half of the sample (51.9%) had an annual household income >$75 000. Mean BMI was 35.4 kg/m^2^ (SD 9.1) and 22.3% had completed MBS. Self‐reported medical conditions were highly prevalent and included high blood pressure (43.8%), hyperlipidaemia (32.8%), sleep apnea (28.7%), diabetes (28.4%), asthma (20.7%), heart disease (9.0%) and active cancer treatment (3.1%). A total of 17 patients (2.9%) reported they had tested positive for SARS‐CoV‐2, while more patients (13.5%) reported having COVID‐19 symptoms. Most (82.7%) patients chose to stay at home despite lifting of the COVID‐19 stay‐at‐home orders, while 72.2% reported only leaving their homes for necessities, followed by 41.4% who went outside for walks or exercise (Table 1).

**TABLE 1:** Patient demographic and medical information, COVID‐19 and obesity study phase II (n = 589)

Variables	Total (n = 589)
Male, n (%)	98 (16.7)
Age, mean (SD)	53.6 (12.8)
Race, n (%)	
Non‐Hispanic white	314 (54.5)
Non‐Hispanic black	124 (21.5)
Hispanic	114 (19.8)
Other	24 (4.2)
Education, n (%)	
Some high school	6 (0.2)
High school graduate	41 (7.0)
Some college of technical school	200 (34.1)
College graduate	339 (57.8)
Annual household income, n (%)	
<$25 000	59 (10.1)
$25 000‐49 999	99 (17.0)
$50 000‐74 999	122 (21.0)
≥75 000	302 (51.9)
BMI, mean (SD)	35.4 (9.1)
Completed metabolic and bariatric surgery, n (%)	130 (22.3)
Medical conditions, n (%)	
Active cancer treatment	18 (3.1)
Asthma	122 (20.7)
Diabetes	167 (28.4)
Heart disease	53 (9.0)
High blood pressure	258 (43.8)
High cholesterol/hyperlipidaemia	193 (32.8)
Sleep apnea	169 (28.7)
Test positive for COVID‐19, n (%)	17 (2.9)
Had COVID‐19 symptoms, n (%)	79 (13.5)
Staying at home since COVID‐19 order has been lifted, n (%)	483 (82.7)
Level of quarantine, n (%)	
Not going outside at all	21 (3.6)
Going outside for walks or exercise	244 (41.4)
Going outside for necessities (food, medications)	425 (72.2)
Visiting close family/friends	147 (25.0)
Going to work	80 (13.6)
Attending religious services	17 (2.9)
Attending parties/large social functions	1 (0.2)
Going out as normal	3 (0.5)
Lost job since COVID‐19, n (%)	39 (6.7)
Drug use during past 30 d, n (%)	
Sedatives or tranquillisers	56 (9.5)
Painkillers	143 (24.3)
Marijuana/Cannabis	21 (3.6)
Stimulants	6 (1.0)
Poly drug use, n (%)^a^	
≥2 drugs	34 (5.8)
≥3 drugs	3 (0.5)
Alcohol use (days) during past 30 d, mean (SD)	3.7 (6.2)
Current cigarette use, n (%)	19 (6.0)
Increased the use of any recreational substances since the COVID‐19 stay‐at‐home orders, n (%)	57 (9.8)
Reason for the increase in recreational substance use, n (%)	
Stress	41 (71.9)
Anxiety	37 (64.9)
Depression	23 (40.4)
Boredom	28 (49.1)
Coping mechanism	29 (50.9)
To keep weight down	2 (3.5)

^a^ Two drugs combination includes sedatives or tranquillisers and painkillers; sedatives or tranquillisers and marijuana/cannabis; sedatives or tranquillisers and stimulants; painkillers and marijuana/cannabis; painkillers and stimulants; marijuana/cannabis and stimulants; Three drugs combination includes: sedatives or tranquillisers and painkillers and marijuana/cannabis; sedatives or tranquillisers and painkillers and stimulants; No participants reported using all four drugs.

During the preceding 30 days, almost a quarter (24.4%) of patients reported the use of opioids, while almost 10% (9.5%) reported the use of sedatives or tranquillisers, marijuana/cannabis (3.6%), or stimulants (1.0%). On average, patients consumed alcohol‐containing drinks on 3.7 (SD 6.2) days per month and 23.6% of the sample had alcohol at least once a week (>4 days during the past 30 days). 6% of the sample reported current tobacco use. Notably, about 10% of our sample reported increasing the use of any recreational substances since the start of COVID‐19 stay‐at‐home orders, and the primary reasons for this increased use were stress (71.9%) and anxiety (64.9%) (Table 1).

The majority (68.6%) of the sample reported that it was more difficult to achieve their weight loss goals during the pandemic, while 23.0% reported no impact. About a half (50.3%) reported a decreased amount of time for exercise, and 53.6% reported a decrease in intensity of exercise. Significantly more substance non‐users reported increasing exercise intensity than substance users (7.2% vs 3.3%, *P* = .029) (Table 2). Slightly more than a third (39.9%) of the sample reported grocery shopping once a week, while just less than a third (31.1%) reported shopping 1 to 2 times a month. More than half of the sample reported an increase in stockpiling food. Well over half (61.5%) reported healthy eating was more challenging, and 61.3% reported stress eating. More than a half reported cooking more often (55.9%), while less than one‐third (27.9%) reported baking more. Food insecurity was described by 21.6% of the sample and 12.1% reported skipping meals. Over half (57.5%) reported their frequency of leaving their home did not change after the stay‐at‐home orders were lifted. None of these reported behaviour changes were significantly different by substance use group.

**TABLE 2:** Change of weight related‐behaviours and psychological factors post‐COVID‐19 lockdown orders by substance use, COVID‐19 and obesity study phase II

		N (%)			
		Total	Substance users (n = 285)	Non‐users (n = 304)	*P* value^l^
Weight loss goal^a^	Easier to achieve	49 (8.4)	20 (3.4)	29 (5.0)	.219
	Not affect	134 (23.0)	59 (10.1)	75 (12.9)	
	Harder to achieve	400 (68.6)	203 (34.8)	197 (33.8)	
Exercise time^b^	Decreased	295 (50.3)	149 (25.4)	146 (24.9)	.278
	Unchanged	123 (20.9)	62 (10.6)	61 (10.4)	
	Increased	101 (17.2)	40 (6.8)	61 (10.4)	
	Do not exercise	68 (11.6)	33 (5.6)	35 (6.0)	
Exercise intensity^c^	Decreased	277 (53.6)	137 (26.5)	140 (27.1)	.029
	Unchanged	186 (36.0)	96 (18.6)	90 (17.4)	
	Increased	54 (10.4)	17 (3.3)	37 (7.2)	
Food shopping frequency^d^	Never/home delivery	94 (16.2)	48 (8.3)	46 (7.9)	.802
	1‐2 times/mo	180 (31.1)	87 (15.0)	93 (16.1)	
	1 time/wk	231 (39.9)	111 (19.2)	120 (20.7)	
	≥2 times/wk	74 (12.8)	40 (6.9)	34 (5.9)	
Stockpile food^e^	Less	17 (2.9)	8 (1.4)	9 (1.6)	.520
	Unchanged	251 (43.4)	115 (19.9)	136 (23.5)	
	More	310 (53.6)	157 (27.1)	153 (26.5)	
Follow health diet plans^f^	Easier	56 (9.7)	19 (3.3)	37 (6.4)	.056
	Unchanged	167 (28.8)	80 (13.8)	87 (15.0)	
	More challenging	356 (61.5)	182 (31.4)	174 (30.1)	
Stress eat more^e^	Yes	354 (61.3)	179 (31.0)	175 (30.3)	.239
	No	224 (38.7)	102 (17.7)	122 (21.1)	
Cooking activity^g^	Less	84 (14.6)	43 (7.5)	41 (7.1)	.767
	Unchanged	170 (29.5)	79 (13.7)	91 (15.8)	
	More	322 (55.9)	157 (27.3)	165 (28.6)	
Baking activity^h^	Less	88 (15.2)	41 (7.1)	47 (8.1)	.915
	Unchanged	330 (56.9)	160 (27.6)	170 (29.3)	
	More	162 (27.9)	80 (13.8)	82 (14.1)	
Cannot afford to eat balanced meals^i^	Often	33 (5.8)	13 (2.3)	20 (3.5)	.178
	Sometimes	85 (14.8)	37 (6.5)	48 (8.4)	
	Never	449 (78.4)	221 (38.6)	228 (39.8)	
Going out frequency since the stay‐at‐home orders have been lifted^a^	Less	150 (25.7)	74 (12.7)	76 (13.0)	.840
	No difference	335 (57.5)	158 (27.1)	177 (30.4)	
	More	98 (16.8)	49 (8.4)	49 (8.4)	
Difficulty falling asleep^j^	Yes	367 (62.8)	191 (32.7)	176 (30.1)	.032
	No	217 (37.2)	93 (15.9)	124 (21.2)	
Anxiety^a^	Yes	376 (64.5)	191 (32.8)	185 (31.7)	.142
	No	207 (35.5)	92 (15.8)	115 (19.7)	
Depression^k^	Yes	461 (81.4)	230 (40.6)	231 (40.8)	.261
	No	105 (18.6)	46 (8.1)	59 (10.4)	

^a^ N_missing_ = 6.

^b^ N_missing_ = 2.

^c^ N_missing_ = 72.

^d^ N_missing_ = 21.

^e^ N_missing_ = 11.

^f^ N_missing_ = 10.

^g^ N_missing_ = 13.

^h^ N_missing_ = 9.

^i^ N_missing_ = 16.

^j^ N_missing_ = 5.

^k^ N_missing_ = 20.

^l^ Chi‐square test.

Table 2 also shows a majority of the sample reported difficulty falling asleep (62.8%), anxiety (64.5%) and depression (81.4%). Substance use groups differences were significant for difficulty falling asleep (substance users 32.7% vs non‐users 30.1%, *P* = .032), but not for anxiety or depression.

After controlling for key demographics, education, BMI, and COVID‐19 infection, patients who reported stockpiling food more (adjusted Odds Ratio [aOR] 1.50, 95% CI 1.03‐2.18), healthy eating more challenging (aOR 1.47, 95% CI 1.01‐2.16), difficulty falling asleep (aOR1.64, 95% CI 1.14‐2.34) and anxiety (aOR 1.47, 95% CI 1.01‐2.14) were significantly more likely to report substance use compared with non‐users (Table 3).

**TABLE 3:** Crude and adjusted odds ratio for substance use by weight‐related behaviours and psychological factors

Variables	Crude odds (95% CI)^a^	*P* value^a^	Adjusted odds (95% CI)^b^	*P* value^b^
Stockpile food				
Less or unchanged	1.0 (ref)	—	1.0 (ref)	—
More	1.20 (0.87‐1.66)	.269	**1.50 (1.03–2.18)**	**.035**
Follow healthy eating pattern				
Easier or unchanged	1.0 (ref)	—	1.0 (ref)	—
More challenging	1.29 (0.93‐1.81)	.131	**1.47 (1.01–2.16)**	**.048**
Stress eat more				
Yes	1.22 (0.88‐1.71)	.239	1.34 (0.92‐1.99)	.129
No	1.0 (ref)	—	1.0 (ref)	—
Cooking activity				
Less	1.21 (0.72‐2.04)	.548	1.38 (0.76‐2.52)	.427
Unchanged	1.0 (ref)	—	1.0 (ref)	—
More	1.10 (0.76‐1.59)	.987	1.23 (0.81‐1.88)	.801
Baking activity				
Less	0.93 (0.58‐1.49)	.69	1.11 (0.64‐1.92)	.681
Unchanged	1.0 (ref)	—	1.0 (ref)	—
More	1.04 (0.71‐1.51)	.71	0.98 (0.65‐1.49)	.757
Going out frequency since the stay‐at‐home orders have been lifted				
Less often	1.09 (0.74‐1.60)	.880	1.04 (0.67‐1.61)	.810
No difference	1.0 (ref)	—	1.0 (ref)	—
More often	1.12 (0.71‐1.76)	.755	0.97 (0.57‐1.64)	.845
Weight‐loss goal				
Easier to achieve	0.88 (0.45‐1.70)	.386	1.02 (0.49‐2.10)	.688
Unchanged	1.0 (ref)	—	1.0 (ref)	—
More difficult to achieve	1.31 (0.88‐1.94)	.088	1.35 (0.87‐2.10)	.180
Exercise time				
Decreased	1.0 (0.66‐1.53)	.298	1.08 (0.69‐1.69)	.363
No difference	1.0 (ref)	—	1.0 (ref)	—
Increased	1.21 (0.69‐2.15)	.072	0.8 (0.45‐1.41)	.317
Do not exercise	0.93 (0.51‐1.68)	.790	0.98 (0.53‐1.83)	.912
Exercise intensity				
Decreased	0.92 (0.63‐1.33)	.099	1.01 (0.68‐1.49)	.142
Unchanged	1.0 (ref)	—	1.0 (ref)	—
Increased	**0.43 (0.23‐0.82)**	**.010**	0.54 (0.28‐1.06)	.056
Difficulty falling asleep				
Yes	**1.45 (1.03‐2.03)**	**.032**	**1.64 (1.14–2.34)**	**.007**
No	1.0 (ref)	—	1.0 (ref)	—
Anxiety				
Yes	1.29 (0.92‐1.81)	.152	**1.47 (1.01‐2.14)**	**.047**
No	1.0 (ref)	—	1.0 (ref)	—
Depression				
Yes	1.28 (0.83‐1.96)	.261	1.36 (0.87‐2.13)	.182
No	1.0 (ref)	—	1.0 (ref)	—

^a^ Crude logistic regression.

^b^ Also adjusted for age, gender, race, education, BMI, COVID‐19 infection.

Table 4 demonstrates the crude and adjusted odds ratios using increased substance use during the COVID‐19 pandemic as the dependent variable. After adjustment, those who reported increased stress eating had 6 times higher odds of increasing substance use (aOR 6.34, 95% CI 2.41‐16.65). Subsequently, patients who reported more difficulty achieving their weight‐loss goal (aOR 4.99, 95% CI 1.74‐14.35), depression (aOR 4.42, 95% CI 1.33‐14.65), healthy eating more challenging (aOR 3.47 95% CI 1.65‐7.31), anxiety (aOR 3.24, 95% CI 1.45‐7.21), decreased exercise time (aOR 2.69, 95% CI 1.09‐6.67), difficulty falling asleep (aOR 2.23, 95% CI 1.13‐4.37), and food stockpiling (aOR 2.07, 95% CI 1.12‐3.83) had 2 to 5 times higher odds of increasing substance use vs those who did not report increasing substance use.

**TABLE 4:** Crude and adjusted odds ratio for increased substance use by weight‐related behaviours and psychological factors

Variables	Crude odds (95% CI)^a^	*P* value^a^	Adjusted odds (95% CI)^b^	*P* value^b^
Stockpile food				
Less or unchanged	1.0 (ref)	—	1.0 (ref)	—
More	**2.11 (1.66‐3.82)**	**.014**	**2.07 (1.12–3.83)**	**.001**
Follow healthy eating pattern				
Easier or unchanged	1.0 (ref)	—	1.0 (ref)	—
More challenging	**3.59 (1.73‐7.47)**	**<.001**	**3.47 (1.65–7.31)**	**.048**
Stress eat more				
Yes	**6.03 (2.54‐14.31)**	**<.001**	**6.34 (2.41–16.65)**	**<.001**
No	1.0 (ref)	—	1.0 (ref)	—
Cooking activity				
Less	**2.48 (1.08‐5.71)**	**.036**	1.94 (0.70‐5.35)	.308
Unchanged	1.0 (ref)	—	1.0 (ref)	—
More	1.42 (0.71‐2.84)	.711	1.54 (0.72‐3.31)	.762
Baking activity				
Less	0.55 (0.22‐1.34)	.52	0.56 (0.19‐1.68)	.741
Unchanged	1.0 (ref)	—	1.0 (ref)	—
More	0.54 (0.27‐1.10)	.43	0.46 (0.21‐1.01)	.278
Going out frequency since the stay‐at‐home orders have been lifted				
Less often	1.17 (0.61‐2.25)	.908	1.46 (0.71‐2.99)	.568
No difference	1.0 (ref)	—	1.0 (ref)	—
More often	1.48 (0.72‐3.02)	.376	1.41 (0.61‐3.24)	.708
Weight‐loss goal				
Easier to achieve	0.67 (0.07‐6.12)	.254	0.61 (0.07‐5.68)	.217
Unchanged	1.0 (ref)	—	1.0 (ref)	—
More difficult to achieve	**4.82 (1.71‐13.6)**	**.002**	**4.99 (1.74–14.35)**	**.002**
Exercise time				
Decreased	2.72 (1.12‐6.63)	.054	**2.69 (1.09‐6.67)**	**.036**
No difference	1.0 (ref)	—	1.0 (ref)	—
Increased	2.62 (0.87‐7.91)	.233	1.17 (0.37‐3.69)	.283
Do not exercise	1.44 (0.47‐4.43)	.508	2.56 (0.83‐7.89)	.201
Exercise intensity				
Decreased	1.39 (0.73‐2.67)	.278	1.54 (0.78‐3.04)	.204
Unchanged	1.0 (ref)	—	1.0 (ref)	—
Increased	0.91 (0.29‐2.86)	.629	0.94 (0.29‐3.09)	.627
Difficulty falling asleep				
Yes	**2.41 (1.24‐4.66)**	**.009**	**2.23 (1.13–4.37)**	**.020**
No	1.0 (ref)	—	1.0 (ref)	—
Anxiety				
Yes	**3.67 (1.70‐7.91)**	**<.001**	**3.24 (1.45–7.21)**	**.004**
No	1.0 (ref)	—	1.0 (ref)	—
Depression				
Yes	**4.47 (1.37‐14.60)**	**.013**	**4.42 (1.33–14.65)**	**.015**
No	1.0 (ref)	—	1.0 (ref)	—

^a^ Crude logistic regression.

^b^ Also adjusted for age, gender, race, education, BMI, COVID‐19 infection.

**FIGURE 1:**
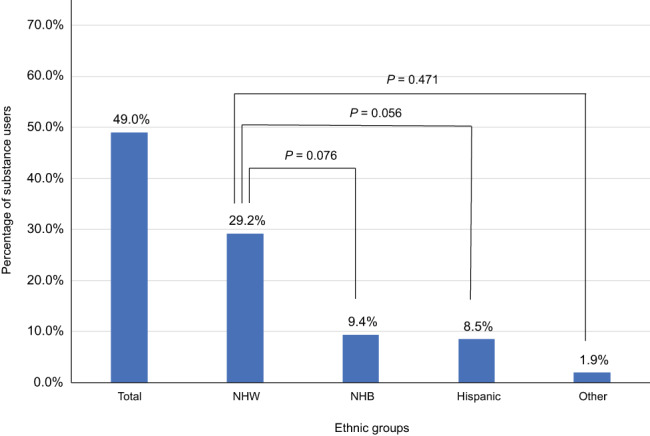
Substance use by different ethnic groups since COVID‐19 stay‐at‐home order has been lifted. *P* values were generated from Pearson chi‐square tests

Among our sample, 29.2% of substance users were NHW followed by 9.4% NHB, 8.5% Hispanic, and 1.9% other races. Using NHW as the reference group, no statistically significant difference was detected among ethnic groups. In total, nearly a half (48.4%) of our sample reported substance use (Figure 1).

## 5 DISCUSSION

Data from this study clearly demonstrate that the COVID‐19 pandemic has continued to impact people with obesity by negatively influencing mental health and facilitating substance use. It is concerning that a significant proportion of participants have turned to substance use and/or increased substance use during the pandemic to help manage their stress and mental health. It has been well established in the literature that there is a multi‐faceted relationship between substance use disorders and mental health conditions, which is thought to be related to the brain's response to chronic stress.^20^ The 2019 National Survey on Drug Use and Health showed that these conditions are prevalent in society and nearly 4% of Americans reported both a substance use disorder and a mental health condition in 2019, which was an increase from 3.3% in 2015.^21^ Given the increasing prevalence of coexistent mental health conditions and substance use in society, our findings raise concerns that people with obesity, who are already at risk of developing these conditions separately, will be at an even greater risk due to the stress of the COVID‐19 pandemic. In addition, participants who reported difficulty falling asleep and anxiety were significantly more likely to report substance use, which aligns with data from the NSDUH.

Although there is limited literature to date on the relationship between substance use disorder and obesity, it is understood that life stress can increase the risk of developing substance use disorders and that there is a bidirectional relationship between stress and obesity.^22^ Our findings raise concerns that the COVID pandemic has increased stress levels, particularly in people with obesity, leading to maladaptive coping behaviours such as increased stress‐eating and food stockpiling, which make achieving weight loss goals more difficult and were all associated with a higher likelihood of increasing substance use. This association is thought to be related to the parallels that exist between eating behaviours and addictive behaviours found in substance dependence based on the shared behavioural features and dopaminergic reward pathways in the brain.^8^, ^23^

Studies show that stress in early life increases the risk of developing obesity through complex mechanisms that are not entirely understood.^24^ Placing this and our findings in the context of rising rates of obesity, suggests that we need to integrate trauma informed care into both primary care and obesity care programs, especially in the setting of additional stressors like the COVID‐19 pandemic. For people with obesity in our study, the added psychosocial and financial stress of the pandemic appear to be related to new and increasing use of substances like alcohol, recreational drugs and prescription medications as coping mechanisms.

Earlier in the pandemic, studies reported the initial impact of mandated stay‐at‐home orders, stress and disruption for people with obesity. It is well known that the COVID‐19 pandemic has made weight loss more difficult for many by facilitating obesogenic behaviours that include decreased exercise, increased stockpiling of food and stress eating.^25^ However, there is not yet sufficient data on the long‐term effects of the COVID‐19 pandemic in people with obesity. This study demonstrates that adults with obesity continue to engage in the same behaviours and struggle with mental health challenges, even after lockdown orders were lifted. Therefore, it will be important to develop community‐level interventions, including health communication and engagement strategies, targeting these vulnerable groups to address compounded mental health issues and psychosocial stressors that may drive a wave of substance use disorders for the duration of the COVID‐19 pandemic and beyond.

### 5.1 Study limitations and strengths

There are several limitations to this study that should be mentioned. First, this was a sample of convenience which can produce selection bias. Respondents were enrolled from an academic medical centre's weight management program and two MBS clinics and were primarily college‐educated NHW women with an average age of 51 years, average BMI of 40.6 kg/m^2^ and an annual income of ≥$75 000. This study may not be generalizable to a clinic, or general population with healthy weight. It may also not be generalizable to other populations and as a result may not accurately assess the burden of COVID‐19 on obesity‐related health and behaviours in lower socioeconomic status and/or ethnic minority populations who are disproportionately more affected by obesity and COVID‐19.^26^ Participants were established weight management patients with secured health insurance, which is not representative of the average American challenged with obesity in which <2% receive anti‐obesity medications^27^ and <1% undergo MBS.^28^ Another limitation is that behavioural changes were based on the patient's own perception at the time of the survey rather than on quantifying these behaviours (such as exercise time decreased from 120 to 60 min/wk). It also did not capture information on attitudes about health and psychosocial health in particular. Furthermore, we are inferring that an increase in behaviours that favour weight gain during COVID‐19 would lead to worsening obesity but do not present longitudinal causal effect data on changes in weight. Strengths of the study include the capture of important information to inform comprehensive, future health care for patients with obesity. Certainly health care access should be maintained in some way during these periods moving forward especially if the pandemic should cause future stay‐at‐home orders as there are implications for long term health as shown from this stay‐at‐home situation.

## 6 CONCLUSIONS

The COVID‐19 pandemic is having a durable negative effect on the health behaviours and mental health of people with obesity. In particular, this group is reporting significant use of substances, increases in anxiety, depression and difficulty sleeping, which are associated with maladaptive eating and physical activity behaviours. These findings can inform healthcare providers on developing screening and intervention programs for this at‐risk population.

## CONFLICTS OF INTEREST

No conflict of interest was declared.
